# The synergy between leadership, work regime, and quality-of-life

**DOI:** 10.3389/fpsyg.2025.1672785

**Published:** 2025-10-10

**Authors:** Vasco José, Ana Palma-Moreira, Manuel Au-Yong-Oliveira

**Affiliations:** ^1^Faculdade de Ciências Sociais e Tecnologia, Universidade Europeia, Lisboa, Portugal; ^2^INESC TEC, Porto, Portugal; ^3^GOVCOPP, DEGEIT, University of Aveiro, Aveiro, Portugal

**Keywords:** leadership, work regime, quality of life, remote work, flexible work

## Abstract

**Introduction:**

The primary objective of this research was to investigate the relationship between leadership and quality of life and whether this association is moderated by the work regime (remote, hybrid, or in-person). Additionally, we aimed to investigate the relationship between work regime and quality of life, as well as the mediating effect of leadership on this relationship.

**Methods:**

A total of 231 individuals working under different work regimes (in-person, hybrid and remote) participated in this study. This study followed a quantitative methodology.

**Results:**

Leadership has a positive and significant association with the perception of quality-of-life. The work regime has a significant effect on the perception of quality-of-life and moderates the relationship between transformational leadership and the perception of quality-of-life. The work regime has a significant impact on the perception of leadership. Leadership has a mediating effect on the relationship between the work regime and the perception of quality of life.

**Discussion:**

This study demonstrates that technology can be an ally in increasing individual well-being, provided it is accompanied by effective leadership practices tailored to the adopted regime. Employees’ perception of quality of life emerges not only because of the conditions adopted, but also from a synergy involving the context (work regime) and management capable of leading in a manner appropriate to the remote or hybrid context.

## 1 Introduction

In an increasingly globalized world, companies must keep pace with technological developments (Khanom, 2023) while also preserving their human capital by retaining their employees, so that they can succeed and compete in their market (Lima, 2020) through sustainable strategies. In other words, the ability to continuously adapt is decisive for an organization to thrive, both in terms of technology and, in conjunction with this, by aligning its operations with the preferences and needs of its employees, thereby ensuring its long-term smooth functioning.

The well-being of any human being is vital to their proper functioning, whether cognitive or physical, and the work routine they have in their life can directly affect their quality of life and the way each person feels and acts towards other parts of their life, not just their professional life. However, in the context of work, employees need to have their needs met in order for their work performance to be effective and contribute to the organization’s growth (Dewi and Ikwan, 2024).

As the years pass, it has become increasingly important to focus on mental health and its significant impact on a person’s life, particularly in their professional life, which is important for maintaining good health, given that they spend a third of their day at work. Here, both leadership (Hermanto et al., 2024) and the work regime (remote, hybrid, and in-person) play a critical role in elucidating the relationship between a person’s quality of life.

Leadership plays a crucial role in shaping how individuals perceive their daily work life and their overall satisfaction (Hermanto et al., 2024), which in turn affects their quality of life. The type of leadership with the most significant impact on satisfaction is related to innovation and is called Transformational Leadership (Butt et al., 2019; Nanjundeswaraswamy and Swamy, 2018).

The work regime is associated with a better quality of life, considering the lives and preferences of workers (Lehto, 2023). Remote working, for example, is associated with longer sleep times, which in the long term translates into lower levels of fatigue (Wells et al., 2023), as well as better use of time during the day, avoiding commuting (Smite et al., 2022a, 2022b), which will bring more quality of life. However, it may be related to higher levels of physical inactivity (Wells et al., 2023) and isolation, which can impact work performance (Smite et al., 2022a, 2022b) in tasks that require more brainstorming and teamwork. According to Petitta and Ghezzi (2025), the potential disadvantages of flexible working arrangements should be considered, as well as the need to organize remote work according to the wishes and needs of employees.

This study aims to provide both the literature and organizations with new guidelines related to leadership, work arrangements, and employee quality of life, since few studies have focused on the synergy between these three variables.

This study aims: to study the effect of leadership on quality of life and whether this relationship is moderated by the work regime (in-person, hybrid, and remote).; to study the effect of the work regime (in-person, hybrid, and remote) on the participants’ quality of life and whether this relationship is mediated by leadership.

## 2 Literature review

### 2.1 Quality of life

The concept of quality of life has evolved. Previously, more emphasis was placed on what one had rather than what one is or thinks one is, as is considered today. It is influenced by external factors with which a person comes into contact and lives (Owczarek, 2010) and is not something that remains constant or is always perceived in the same way. It is, therefore, a subjective concept for measuring an individual’s well-being, which can be influenced by external factors encompassing the needs of various dimensions, such as health (physical and mental), comfort, emotions, social aspects, and material possessions (Dalia and Ruzevicius, 2014).

In the context of organizations and the quality of life of workers, many factors can shape it, which is why the term Quality of Work Life (QWL) emerges as a measure of the well-being of workers towards their work and, consequently, towards the organization. This work-related term is often associated with higher levels of stress when it is at lower levels (Melandari and Jannah, 2025). Stress, in turn, negatively influences how employees perform their tasks, as well as their perception of their quality of work life (Dewi and Ikwan, 2024), which in turn conditions organizational success. It is essential to implement strategies and policies to reduce stress levels (and other negative feelings that accompany it) to minimize this problem. Some relevant examples for this study are highlighted below (excluding motivation through salary increases, which can increase the perception of quality of life): employee retention policies that include training to prevent turnover (Pimenta de Brito et al., 2025); adjusting schedules to improve sleep quality (Kim et al., 2024) and work flexibility that encompasses work-life balance (Maidment et al., 2024).

Some aspects to consider that impact on employees’ perception of quality of life include the feeling of belonging, tasks performed, career growth, and the work environment (Suyunova et al., 2024).

For the context of this study, the focus is more on work flexibility, which is where hybrid and remote work comes in, as they have been showing positive results not only for employees but also for organizations. By having more time for their personal lives, employees become happier, more satisfied, and more willing, which will have positive consequences for the results of organizations, allowing for a better balance between their daily responsibilities, whether personal or work-related (Shandu et al., 2024).

Another relevant point to mention about the impact of remote work on quality of life is that it softens or removes any geographical barriers that may exist (Braesemann et al., 2022). By having access to all the necessary information for remote work, organizations also benefit from not having to pay for physical space, thereby reducing expenses by choosing to work remotely (Choudhury and Foroughi, 2021).

The synergy created by the benefits that remote work and flexible hours provide for both employees and organizations is evident.

### 2.2 Leadership

The importance of leadership in achieving results for an organization is worth studying, since the type of leadership (or style) practiced in conjunction with other employees (or subordinates) and its synergy with everyone’s personality has an impact on productivity and, consequently, on their performance (Pasaribu et al., 2022). Each employee will respond differently to each leadership style practiced, with some being more helpful than others, considering not only their way of being, interpreting, and acting (Putri et al., 2020), but also the organizational civility that they may have at higher levels than others. The situational leadership style plays a fundamental role in adapting to employees and unexpected situations (Pasaribu et al., 2022).

There is therefore an evolution in the way leadership is studied, in which it is no longer governed solely by delegating, monitoring, and coordinating tasks with the employees for whom the leader tends to be hierarchically responsible, but rather a whole spectrum where the role of leader is found and coexists with everything around them (Avolio et al., 2009), as well as the role of mediator between the needs and functions of other employees (Passadas, 2021), regardless of their location, with virtual management and communication skills, especially in remote work (Nogueira and Patini, 2012).

Leadership can therefore be a significant factor in the quality of life of any employee in a company and its overall results (Katili et al., 2021), as management can influence other employees under its authority (Espírito Santo, 2022). Therefore, this mutual relationship can provide numerous benefits that can contribute not only to the employee (contributing to their satisfaction) but also to the organization, though agreements between both parties (Hornung et al., 2011).

Framing leadership within the objective under study, the styles considered most suitable for remote companies are generally considered to be leadership focused on interpersonal relationships, supportive leadership, and, frequently mentioned in these cases, transformational leadership, (Lundqvist et al., 2022) – which, through its creativity and innovation, manages to maintain commitment to employees, ensuring positive changes in an organization and providing desired results (Katili et al., 2021). Transactional leadership is also relevant in remote working contexts (Kairupan, 2023; Dong, 2023), particularly in terms of employee productivity.

Leadership members should possess knowledge that enables them to coordinate teams, work closely with them, and decision-making skills to handle complex situations. Another important feature to highlight is the potential for creativity that every leader must possess when working in modern and/or remote work environments, which calls for innovation. For remote work to function well, leadership must also be very inclusive and open-minded, in the sense of adapting to diverse cultures and different personalities of workers around the world, for example by building virtual work environments (Globalization Partners, 2023), as well as working with different time zones (Henke et al., 2022) – where there must be a solid foundation of trust in the team(s) responsible for carrying out the task(s) on the part of leadership, as well as high levels of responsibility and kindness on the part of employees (McCrae and Costa, 1999; Siegl, 2021).

#### 2.2.1 Leadership and quality of life

As mentioned above, leadership has an impact on employee commitment (Pasaribu et al., 2022), which in turn influences their productivity, willingness, and, consequently, their perception of how work affects the quality of their lives. It is also partly responsible for meeting the needs of those being led (Passadas, 2021), leading to improved job performance. It is therefore also the responsibility of leadership to adopt strategies to retain employees, impacting on their motivation, performance, and quality of life at work (Adams, 2024), such as constant transparency in communication, demonstration of career progression, and balance between personal and professional life, also contributing to a reduction in employee turnover. Some companies are already investing in the health management of their employees through leadership, which acts as a bridge between the company and its employees (Dai et al., 2024). This concept is known as Health-Promoting Leadership, which tends to foster a positive relationship between employees and their work.

According to Herzberg (1964) in his Two-Factor Theory, these strategies, initiated by the organization and its leadership, are seen as hygiene factors, whose objective is to reduce or prevent problems. However, they are not sufficient to completely (or almost completely) eliminate turnover or job dissatisfaction that may arise among employees. The other type of factors that exist, called motivational factors, are more intrinsic to everyone and are linked to each person’s personality and beliefs (Oladimeji, 2024), which can create different work environments.

### 2.3 Work regime

Considering that throughout human evolution, work has been a factor of security and important for maintaining quality of life and its possibilities (Cursino, 2024), from reasons of survival to the existence of an economy, to the point of leading to the emergence of Labor Law (Silva, 2014) and, eventually, respective regimes.

Work regimes are akin to systems of control, organization, and interaction related to work, which determine their respective conditions, behavioral expectations for employees, and supervision. Some regimes are stricter and authoritarian, while others are more flexible (Wood, 2022). Additionally, according to Wood (2022), work regimes with greater control have higher turnover rates compared to those with more flexible models. However, these have been evolving, highlighting industrial revolutions and, in more modern times, the impact that technologies have had not only on people’s daily lives but also on shaping the way work and its regimes are approached (Cursino, 2024).

It has become increasingly common to study and advise on more flexible regimes, even though the media, as they ultimately demonstrate not only a positive impact on workers’ well-being and a reduction in occupational stress, but also an increase in autonomy and collaboration between teams (Mache et al., 2020). The COVID-19 pandemic has further impacted on this flexibility (Smollan et al., 2024), during which many workers lost their jobs (Rothstein and Aughinbaugh, 2022), while others had the opportunity to work completely remotely. There was a need for adaptation and resilience not only on the part of workers but also on the part of organizations (Rudolph and Zacher, 2020), calling for organizational support measures, effective communication, as well as virtual courses and meetings to maintain employee commitment to remote work (Kausar et al., 2023). Since then, remote work has expanded to encompass a wide range of areas and positions, which were previously limited to only a few, such as Information Technology (Haubrich and Froehlich, 2020). Currently (in 2025), it is common to find the following three main types of work arrangements: in-person, hybrid, and remote.

#### 2.3.1 Face-to-face work

The face-to-face work regime was considered “traditional” before the COVID-19 pandemic (Bick et al., 2021) and, like the others, has its advantages and disadvantages, which may depend on the tasks to be performed in a specific job (Dingel and Neiman, 2020), such as construction and healthcare, or even on the preferences of employees who want to work in this way. That said, some of the reasons why the face-to-face regime may be preferable for some workers include social interaction, material aspects, or even a sense of duty (Smite et al., 2023), although the norm today (post-pandemic) and in the future tends to be more of a hybrid regime (Tahlyan et al., 2024), as a broader way of meeting the diverse needs of different workers. Other relevant reasons that lead some workers to prefer the face-to-face work regime include subjective issues such as stress management, challenges related to social isolation, and/or access to specific resources that are not available remotely (Smite et al., 2023).

Currently, some companies are losing talent by opting for an entirely in-person regime (Harding, 2024; Smite et al., 2022a, 2022b) and even struggling to fill job vacancies that require full in-person presence (Stillman, 2023), which shows that candidates are currently not interested in a daily routine where most of their time is consumed by work, for a variety of reasons, including long and/or stressful commutes, fewer distractions, work-life balance (Smite et al., 2022a, 2022b), etc.; thus opening up greater possibilities for reducing burnouts (Tahlyan et al., 2024).

Reinforcing the preference for flexibility in working arrangements, Generation Z (born between 1995 and 2010) tend to prefer hybrid or remote work, both because of their ease in dealing with technology throughout their upbringing and because they tend to prioritize their social values, diversity, and social responsibility (Anjum, 2024), not accepting working under conditions they consider too rigid or that prevent them from enjoying their time. In other words, this tends to be a generation of people who prefer to perform tasks that align with their values, based on their principles (Benítez-Márquez et al., 2021; Bellinder, 2024).

#### 2.3.2 Hybrid work

The hybrid work regime is seen as “the best of both worlds.” On the one hand, face-to-face interaction between workers is beneficial, while on the other, it allows for better time management and a work-life balance, already offering some flexibility (Smite et al., 2022a, 2022b). Currently, this is the most popular work arrangement and tends to be the most widely practiced (Tahlyan et al., 2024; Bhat et al., 2023; Alexander et al., 2021) in contexts that allow it, without harming employee productivity or the quality of their work (Bloom et al., 2024). It is relevant to illustrate one of the reasons for this work arrangement by mentioning that, following the COVID-19 pandemic, some professionals in the software field exhibited resistance to returning to their daily office routine (Santos et al., 2024), which spread to other areas of activity (Smite et al., 2022a, 2022b).

This work regime shares both the advantages and disadvantages of the others mentioned, with both being practiced, and can be seen as a win-win for both parties – company and employee – by maintaining the more traditional aspect of physical presence and flexibility through a virtual presence (Bloom et al., 2024), achieving a balance and enabling companies to also achieve partial cost reductions (Choudhury and Foroughi, 2021; Braesemann et al., 2022), for example in facilities.

#### 2.3.3 Remote work

At the end of the 20th century, teleworking was promoted because of the benefits it would bring in terms of reducing road traffic, air pollution and, above all, improving workers’ quality of life (Mokhtarian and Salomon, 1997), addressing various social and organizational failures in large cities (Bailey and Kurland, 2002), where the term “telecommuting” was first used in 1975 by Jack M. Nilles. The goal was to promote a healthy relationship between personal and professional life through organizational and urban decentralization enabled by telecommunications, making this work arrangement more feasible and allowing workers to perform their duties remotely from a physical location (Nilles, 1975). Since then, there has been a prediction that this work regime will become more effective with the advancement of technology.

Although this regime has experienced significant growth following the COVID-19 pandemic in various professional fields, remote work was already being adopted, albeit by a minority (Bailey and Kurland, 2002), particularly in sectors such as information technology, consulting, and financial services (Gajendran and Harrison, 2007).

Of the numerous advantages, two are particularly valuable for workers, which remote work offers better than the other two regimes, which do not allow it so easily: enjoying time by avoiding transportation (and associated costs) to physical spaces (Nogueira Filho et al., 2020; Braesemann et al., 2022) and the elimination of geographical barriers (Braesemann et al., 2022; Choudhury and Foroughi, 2021), providing opportunities for a greater number of workers outside large cities. Another relevant advantage to highlight from a more social and ecological perspective is the reduction in pollution due to the absence of commuting (Knight et al., 2017) for jobs that can be done anywhere on the planet, as already mentioned at the end of the 20th century. For the company, there is also a reduction in costs associated with physical spaces that can accommodate many employees (Choudhury and Foroughi, 2021).

However, for this work regime to be practiced effectively, not only must workers have the essential digital and communication skills, but they must also have the virtual tools that enable them to put them into practice (Lane et al., 2024), even when working with other people in different time zones (Henke et al., 2022). They must also have access to the internet and the necessary equipment to perform their duties remotely (Kothawala et al., 2024). With these requirements in place, the likelihood of effective remote teamwork increases, while the possibility of misunderstandings decreases, making it a functional and productive process. Thus, we mention the online and offline modes of work, which are distinct but necessary for different workers in different locations around the globe to coordinate and perform their tasks promptly (Kim and Oh, 2015), bringing quality of life both to those who perform the tasks and to the company’s business through the concept of Smart Work.

Still, in terms of effectiveness, it is essential for both leadership positions and those hierarchically below them that workers possess digital security competencies to work remotely (Siegl, 2021), to prevent the leakage of sensitive and/or confidential company data. Therefore, all employees working remotely should have a basic understanding of cybersecurity and regularly practice it.

#### 2.3.4 Work regime and quality of life

Quality of life is considered an individual perception, considering each person’s life, personality, and criteria for measuring it (Gill and Feinstein, 1994); therefore, this perception will always be somewhat subjective. It is therefore stated that different working arrangements will provide different perceptions of quality of life to different workers (Lehto, 2023), according to their preferences, and it cannot be said with 100% certainty that one arrangement will be completely better than another.

However, the call for labor flexibility is not random, as it is increasingly discussed in the media and social networks. Companies are constantly adapting to meeting the needs of their employees (as many of them want to have access to full or partial remote work) after the COVID-19 pandemic, without harming their business (Smite et al., 2022a, 2022b).

However, it is possible to highlight some advantages of remote work, regardless of each person’s perception of quality of life, compared to other arrangements (hybrid and face-to-face). These include reduced travel and transportation costs (Nogueira Filho et al., 2020), more time for personal goals unrelated to work (American Psychological Association, 2019), and the total elimination of geographical barriers (Braesemann et al., 2022; Choudhury and Foroughi, 2021). In terms of possible repeat pandemic outbreaks, companies that practice remote working will be even better equipped and able to act more quickly to avoid being adversely affected by the public health impact that could affect everyone involved. Employees would be less exposed to risks and, by working without the need to travel, would be part of a more robust measure in the event of rising unemployment levels (Angelucci et al., 2020).

#### 2.3.5 Leadership, quality of life and work regime

Both leadership and working conditions will have an impact on the quality of life of workers in a given company (Katili et al., 2021; Hornung et al., 2011; Mache et al., 2020), also taking into account factors such as the work environment and the personality of each individual (Putri et al., 2020; Farrell et al., 2024), which will cause their perception of quality of life to change. In other words, it will be the standards (or criteria) that can define much of the quality of life at work.

Leadership will not work in the same way for the three work regimes (face-to-face, hybrid, and remote), nor can anyone take on a leadership role (Clarey, 2022) to manage and meet the needs of those they lead in the three different work modalities. In the context of remote work, leadership should be transformational (Lundqvist et al., 2022), promoting worker autonomy and fostering a vision linked to innovation and evolution, which in turn positively impacts employee job satisfaction (de Melo et al., 2022). Not only should transformational leadership be considered, but transactional leadership should also be considered, which tends to thrive in maintaining high productivity in the daily tasks performed by employees (Kairupan, 2023). Adaptation to the remote context is an ongoing task, regardless of leadership style (Coser et al., 2024). Thus, both transformational and transactional leadership contribute to better organizational performance (Coser et al., 2024; Dong, 2023), including in remote settings. The effectiveness of leadership can also vary depending on the work regime in place (Lundqvist et al., 2022) and may have a greater impact when employees work in person. Not only is it influenced by the regime, but it also can affect or condition, to a certain extent, the perception of quality of life, especially in face-to-face regimes, since this tends to be less impacted by leadership in remote regimes (Coser et al., 2024). We can see not only the differentiating role of leadership depending on the work regime, but also how it affects those being led.

Concerning quality of life and worker preferences about work regimes, face-to-face work tends to lag, along with inflexible working hours (although this does not apply to all employees), with a preference for hybrid and remote work (Tahlyan et al., 2024; Bhat et al., 2023; Smite et al., 2022a, 2022b), the former being the most common today and the latter tending to offer more freedom from various points of view. However, to work remotely effectively, there is a need for greater levels of autonomy, effective communication, conscientiousness, and responsibility, which may not be the most suitable for some workers (Siegl, 2021; Lane et al., 2024). Alongside the two preferred regimes, flexible working hours can significantly impact the quality of life, providing more freedom and better time management (Smite et al., 2022a, 2022b; Yu and Wu, 2021).

### 2.4 Research model and hypotheses

The literature review conducted above leads us to test the association between leadership and quality of life and whether the work regime moderates this relationship. It also leads us to examine the association between the work regime and quality of life and whether this relationship is mediated by leadership.

**Figure 1:**
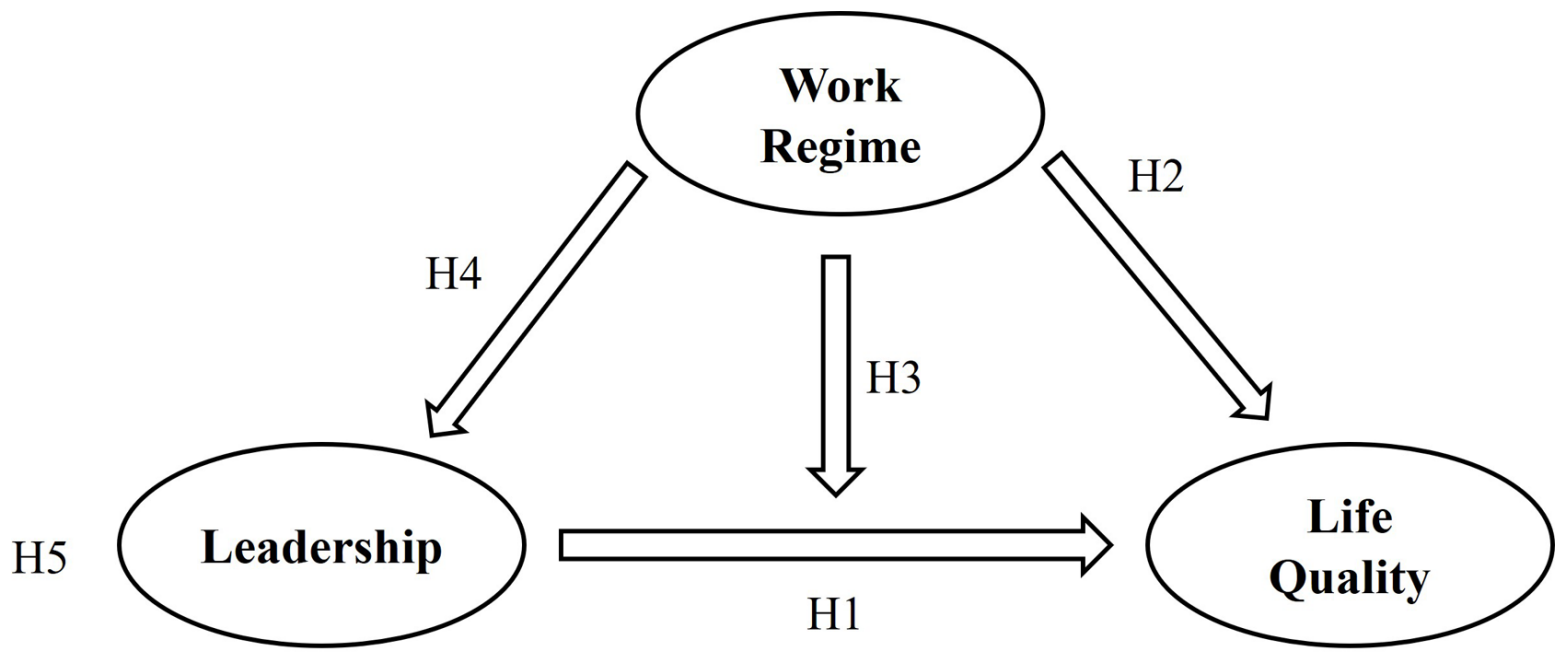
Research model.

The research model presented in Figure 1 summarizes the hypotheses formulated in this study.

*Hypothesis 1*: Leadership has a positive and significant effect on quality of life.

*Hypothesis 2*: The work regime has a significant effect on quality of life.

*Hypothesis 3:* The work regime moderates the relationship between leadership and quality of life.

*Hypothesis 4*: The work regime has a significant effect on leadership.

*Hypothesis 5*: Leadership mediates the relationship between the work regime and quality of life.

## 3 Methods

### 3.1 Data collection procedure

A total of 231 individuals working for companies under various employment regimes participated in this study. The sampling method was non-probabilistic, involving intentional snowball sampling (Trochim, 2000) and convenience sampling. This is also a cross-sectional study.

An online Google Forms questionnaire was distributed through various channels (LinkedIn, Instagram, WhatsApp, and Facebook) via a link (in both Portuguese and English), which individuals accessed and responded to after providing informed consent, thereby relying on their decision-making power. This ensured the confidentiality of each respondent’s answers.

The questions focused on the working conditions of the employee, divided into two scales for leadership and quality of life. In addition to these, sociodemographic questions were also asked to characterize the sample. Data collection took place between January and March 2025.

### 3.2 Participants

The present study has a sample size of 231 participants, the majority of whom are female (61%, *n =* 141), followed by males (38.5%, *n =* 89), and are aged between 21 and 65 years, with an average age of 36.6 years and a standard deviation of 11.3 years. In terms of educational qualifications, most participants held a bachelor’s degree (*n =* 114), corresponding to 49.4%, followed by a master’s degree or higher (*n =* 69), which accounted for 29.9%, and finally, 12th grade or lower (*n =* 48), comprising 20.8%. The vast majority of participants have been with the entity where they perform their duties for 1 to 3 years (*n =* 94), representing 40.7% of the total, followed by less than 1 year and 4 to 6 years (*n =* 86), both representing 18.6%; after these, participants with more than 10 years of seniority (*n =* 33) follow, representing 14.3% of the total, and finally those with between 7 and 10 years (*n =* 18) with 7.8%. Concerning the participants’ employment contracts, 68.8% of them are on permanent contracts, representing the vast majority (*n =* 159), followed by those on fixed-term contracts, corresponding to 13.4% (*n =* 31); followed by those with indefinite contracts (*n =* 29), corresponding to 12.6%; contracts and/or employment relationships designated as “Other” at 3% (*n =* 7) and, finally, self-employed workers, who correspond to only 2.2% (*n =* 5). Regarding employment contracts, 71.4% of all participants are affiliated with the private sector (*n =* 165), 21.6% with the public sector (*n =* 59), and 6.9% with the public/private sector (*n =* 16). In terms of working arrangements, the majority are in-person (*n =* 120), representing 51.9%; followed by those in a hybrid arrangement (*n =* 80), corresponding to 34.6%; and finally, those in a remote arrangement (*n =* 31), corresponding to 13.4%. Thus, regarding remote workdays in a hybrid work regime, the majority is split between three remote workdays or a variable model (*n =* 48), with both options corresponding to 30% each. Next are 4 days of remote work, corresponding to 18.8% (*n =* 15); followed by 2 days of remote work, corresponding to 17.5% (*n =* 14); and finally, a single day of remote work for a minority of 3.8% of the total (*n =* 3).

### 3.3 Data analysis procedure

After collecting, the data was entered into SPSS Statistics 29 software for statistical analysis. The first step was to evaluate the metric qualities of the instruments used in this study.

The validity of the instruments was tested using confirmatory factor analysis in AMOS Graphics 29 software. The procedure followed a “model generation” logic (Jöreskog and Sörbom, 1993). Six fit indices were combined, as recommended by Hu and Hu and Bentler (1999). The fit indices calculated were as follows: chi-square ratio/degrees of freedom (*χ*^2^/gl); Tucker-Lewis Index (TLI); goodness of fit index (GFI); comparative fit index (CFI); root mean square error of approximation (RMSEA); root mean square residual (RMSR). The chi-square/degrees of freedom ratio (*χ*^2^/gl) must be less than 5. The CFI, GFI, and TLI values must be equal to or greater than 0.90. For RMSEA to be considered a good fit, its value must be less than 0.08 (MacCallum et al., 1996). The lower the RMSR value, the better the fit (Hu & Hu and Bentler, 1999). With the data obtained from the confirmatory factor analysis, the reliability construct for each dimension and the convergent validity (as calculated by the AVE value) were assessed. The construct reliability values should be higher than 0.70, and the AVE value should be equal to or higher than 0.50 (Fornell and Larcker, 1981). However, values between 0.40 and 0.50 can be accepted if the Cronbach’s alpha value exceeds 0.70 (Hair et al., 2011). Divergent validity was also calculated.

The internal consistency of the instruments was verified using Cronbach’s alpha coefficient, which ranges from 0 to 1, with negative values being discarded (Hill and Hill, 2002). A coefficient greater than 0.70 was considered the minimum acceptable in organizational research (Bryman and Cramer, 2003). In addition, the sensitivity of the instruments was analyzed by calculating measures of central tendency, dispersion, and distribution for the scale items, which allowed us to assess the normality of the data for all items and scales.

The scale items should have responses distributed across the entire range of the scale, avoiding excessive concentrations at the extremes. Additionally, the limits established for the absolute values of asymmetry (<2) and kurtosis (<7) were respected, as recommended by Finney and DiStefano (2013). After these steps, a descriptive statistical analysis was performed to characterize the sample and the variables studied.

At the beginning of the results, two confirmatory factor analyses were performed to verify whether the theoretical conceptualization that determined the existence of three variables adequately represents the observed data. Discriminant validity was also determined by calculating the square root of the AVE value of each factor, which should be greater than the correlations between the respective factors.

To perform descriptive statistics on the variables under study, t-tests were used for the sample. The association between the variables under study was tested using Pearson correlations. Hypotheses 1 and 5 were tested using simple and multiple linear regressions. For hypotheses 2 and 4, a parametric one-way ANOVA test was performed after verifying the respective assumptions. As for Hypothesis 3, since it involves a moderating effect, Macro Process 4.2, developed by Hayes (2022), was used. A significant level of 0.05 was considered.

### 3.4 Instruments

Leadership was measured using an adapted version of the Multifactor Leadership Questionnaire (Bass, 1985), which was adapted for the Portuguese population by Salanova et al. (2011).

In brief, the scale comprises 28 items, divided into two subscales: transformational leadership and transactional leadership. Transformational leadership consists of five dimensions: idealized attributes (items 1, 2, 3, and 4); idealized behaviors (items 5, 6, 7, and 8); inspirational motivation (items 9, 10, 11, and 12); Intellectual Stimulation (items 13, 14, 15, and 16); Individualized Consideration (items 17, 18, 19, and 20). Transactional leadership consists of two dimensions: Contingent Rewards (items 21, 22, 23, and 24); Management by Active Exception (items 25, 26, 27, and 28). The items are organized on a five-point Likert scale (from 1, “Never” to 5, “Frequently if not always”).

To test the validity of the transformational leadership subscale, a confirmatory factor analysis with five factors was initially performed. Although the fit indices were adequate or very close to adequate values, the factors were strongly correlated (Table 1). A new confirmatory factor analysis with one factor was performed, and adequate fit indices were obtained. Due to these results, in this study, we will consider this instrument as unidimensional (Table 1). Transformational leadership exhibits a composite reliability of 0.96 and an average value of 0.57, indicating good composite reliability and convergent validity. In terms of internal consistency, it has a Cronbach’s alpha of 0.96, which can be considered excellent (Marôco and Garcia-Marques, 2006).

**Table 1:** Results of the confirmatory factor analysis of the leadership scale.

Subscale	Model	*χ*^2^/df	CFI	GFI	TLI	RMSEA	RMSR
Transformational leadership	5 Factors	2.19	0.95	0.87	0.94	0.072	0.054
	1 Factor	1.65	0.98	0.90	0.97	0.053	0.050
Transactional leadership	2 Factors	6.71	0.88	0.90	0.78	0.158	0.158
	1 Factor	2.51	0.99	0.98	0.97	0.081	0.029

Own Source.

For the transactional leadership subscale, a two-factor confirmatory factor analysis was initially performed, but the adjustment indices were not adequate (Table 1). A one-factor confirmatory factor analysis was then performed. Items 25, 27, and 28 were removed because they had low factor loadings. This time, the adjustment indices were adequate (Table 1). Transactional leadership exhibits a composite reliability of 0.80 and an average value of 0.54, indicating good composite reliability and convergent validity. In terms of internal consistency, it has a Cronbach’s alpha of 0.87, which can be considered good (Marôco and Garcia-Marques, 2006).

To measure quality of life, we used the instrument developed by Sirgy et al. (2001) and adapted to the Portuguese population by Sinval et al. (2019). This instrument consists of 16 items distributed across seven dimensions: health and safety needs (items 1, 2, and 3); economic and family needs (items 4, 5, and 6); social needs (items 7 and 8); recognition needs (items 9 and 10); updating needs (items 11 and 12); knowledge needs (items 13 and 14); creativity needs (items 15 and 16). The items are organized on a seven-point Likert scale (from 1, “Absolutely false” to 7, “Completely true”).

To test the validity of the quality-of-life scale, a confirmatory factor analysis was initially performed on seven factors. Although the adjustment indices were adequate, the factors were strongly correlated (Table 2). A new confirmatory factor analysis was performed on one factor, and adequate adjustment indices were obtained. Item 3 had to be removed because it had a low factor weight. Due to these results, in this study, we will consider this instrument unidimensional (Table 2). Quality of life exhibits a composite reliability of 0.92 and an average value of 0.42, indicating good composite reliability. Regarding convergent validity, despite presenting an AVE value of less than 0.50, Cronbach’s alpha value of greater than 0.70, in the view of Hair et al. (2011), suggests that the value presented can be accepted. In terms of internal consistency, it has a Cronbach’s alpha of 0.93, which can be considered excellent (Marôco and Garcia-Marques, 2006).

**Table 2:** Results of the confirmatory factor analysis of the quality-of-life scale.

Model	*χ*^2^/df	CFI	GFI	TLI	RMSEA	RMSR
7 Factors	2.42	0.95	0.90	0.92	0.079	0.146
1 Factor	2.470	0.95	0.90	0.93	0.080	0.133

Own source.

## 4 Results

Two models were tested: one with a single factor and one with three factors. The fit indices for the one-factor model were not adequate (*χ*^2^/df = 4.33; GFI = 0.48; CFI = 0.68; TLI = 0.66; RMSEA = 0.120; RMSR = 0.264). In turn, the fit indices for the three-factor model proved adequate or close to adequate values (*χ*^2^/df = 1.61; GFI = 0.82; CFI = 0.95; TLI = 0.94; RMSEA = 0.051; SMRM = 0.095). It can thus be concluded that theoretical conceptualization, which determines six variables, adequately represents the observed data. The correlations are consistent with the theorized pattern of relationships. On the other hand, it was found that the square root of the AVE values for each factor is greater than the correlation between the respective factors, indicating the existence of discriminant validity.

### 4.1 Descriptive statistics of the variables under study

To perform descriptive statistics on the variables under study, t-tests were used for the sample.

The results indicate that the participants’ responses, both on the transformational leadership subscale and on the transactional leadership subscale, are significantly above the midpoint of the scale (3) (Table 3). Also, about the quality-of-life scale, the participants’ responses are significantly above the midpoint of the scale (4) (Table 3). The participants in this study have a high perception of transformational leadership, transactional leadership, and quality of life.

**Table 3:** Descriptive statistics of the variables under study.

Variável	*t*	*df*	*p*	*d*	Mean	*SD*
Transformational leadership	8.71***	230	< 0.001	0.57	3.50	0.86
Transactional leadership	5.00***	230	< 0.001	0.33	3.30	0.91
Quality-of-life	7.75***	230	< 0.001	0.51	4.64	1.26

***p < 0.001. Own Source.

### 4.2 Association between the variables under study

Pearson correlations were used to study the association between the variables under study.

The results indicate that all variables are positively and significantly correlated with each other (Table 4).

**Table 4:** Association between the variables under study.

Variables	1.1	1.2	2	3
1.1. Transformational leadership	–			
1.2. Transsactional leadership	0.77^***^	–		
2. Quality-of-life	0.59^***^	0.59^***^	–	
3. Work Regime	0.14^*^	0.22^***^	0.28^***^	–

*p < 0.05; ***p < 0.001. Own Source.

The association between quality of life and transformational leadership has the same strength as the association between quality of life and transactional leadership (Table 4). Regarding the work regime, the strongest association is with quality of life and the weakest with transformational leadership (Table 4).

### 4.3 Hypotheses

To test Hypothesis 1, a multiple linear regression analysis was performed after verifying the respective assumptions. This was the appropriate technique, as both the predictor variables and the dependent variable are quantitative, and the sample size is less than 300 participants.

Both transformational leadership (*β* = 0.33, *p <* 0.001) and transactional leadership (*β* = 0.34, *p <* 0.001) have a positive and significant association with quality of life (Table 5). The model explains 39% of the variability in quality of life (Table 5). The model is statistically significant [*F*(2, 228) = 73.59, *p <* 0.001] (Table 5).

**Table 5:** Association between leadership and quality-of-life.

Independent variable	Dependent variable	*F*	*p*	*R^2^_a_*	*β*	*p*
Transformational leadership	Quality-of-life	73.59***	< 0.001	0.39	0.33***	< 0.001
Transactional leadership					0.34***	< 0.001

***p < 0.001. Own Source.

Hypothesis 2 was tested using a one-way ANOVA parametric test, after verifying the respective assumptions. The one-way ANOVA parametric test was chosen because the independent variable was nominal and comprised three groups.

The work regime has a significant effect on quality of life [*F*(2, 228) = 9.54; *p <* 0.001] (Table 6). The quality of life for participants working in person differs significantly from that of participants working remotely or in a hybrid work regime (Table 6).

**Table 6:** Effect of work schedule on quality of life.

Variable	One-way ANOVA		Work Regime. A	Work Regime. B	TuKey HSD	
	F	*p*			Mean Dif. (A-B)	*p*
Quality-of-life	9.54***	< 0.001	Face-to-face	Hybrid	−0.37*	0.021
				Remote	−0.99***	< 0.001

*p < 0.05; ***p < 0.001.

**Figure 2:**
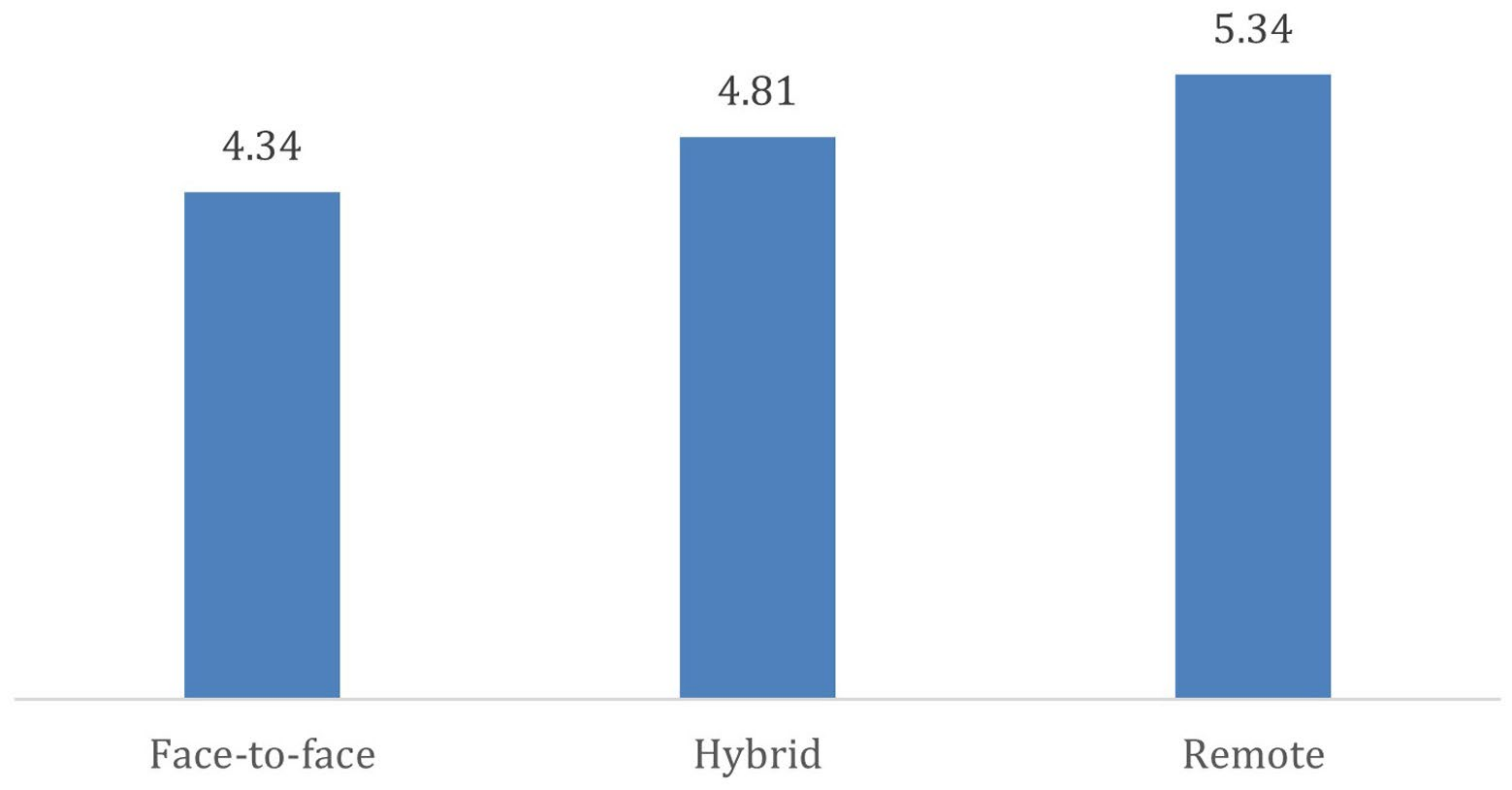
Effect of work regime on quality of life.

Participants who are working remotely reported higher levels of quality of life (Figure 2). On the other hand, participants who are working in person reported lower quality of life levels (Figure 2).

**Figure 3:**
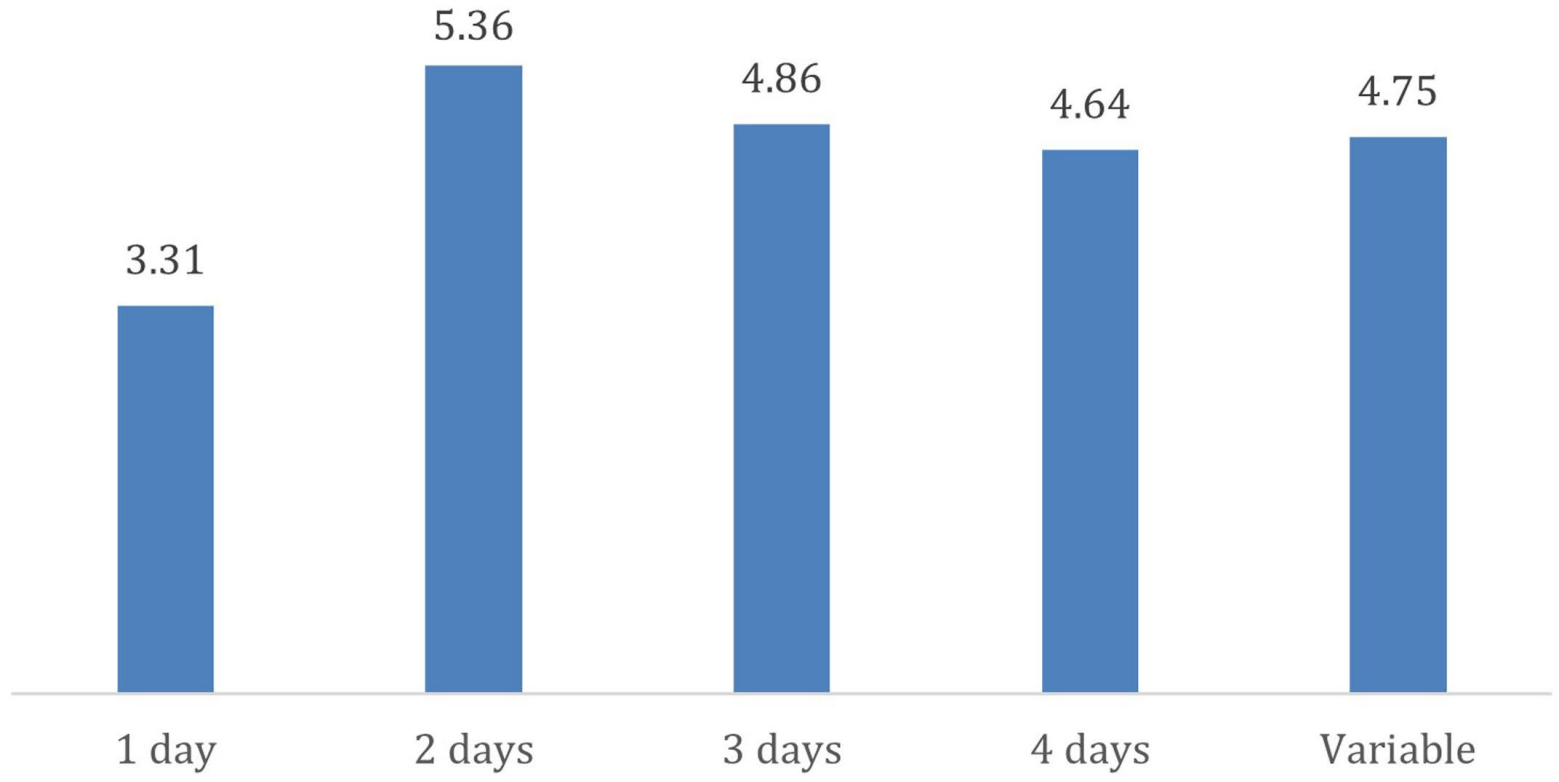
Effect of number of days working remotely per week on quality of life.

Next, we attempted to determine if there were any statistically significant differences in quality of life between participants who work in a hybrid setting, based on the number of days per week they work remotely. The One-Way ANOVA test revealed statistically significant differences [*F*(4, 75) = 2.70; *p* = 0.037]. Participants who work remotely 1 day a week differ significantly from those who work remotely 2 days a week. Among participants in hybrid work arrangements, those who reported higher levels of quality of life are those who work remotely 2 days a week (Figure 3).

To test Hypothesis 3, which assumed a moderating effect, Macro Process 4.2 (Model 1), developed by Hayes (2022), was used, as it is considered the most appropriate method given the size of the sample.

The results indicate that the work regime has a moderate effect on the relationship between transformational leadership and perceived quality of life (B = −0.21; *p* = 0.050) (Table 7).

**Table 7:** Moderating effect results.

Variable	*B*	*SE*	*t*	*p*	95% *IC*
Transformational Leadership → Quality-of-life (R^2^ = 0.40; *p <* 0.001)					
Constant	4.66***	0.07	71.39***	< 0.001	[4.53; 4.78]
Transformational Leadership	0.79***	0.08	10.36***	< 0.001	[0.64; 0.94]
Work Regime	0.40***	0.09	4.23	< 0.001	[0.21; 0.58]
TransfL*WR	−0.21*	0.11	−1.93	0.050	[−0.42; −0.01]
Transactional Leadership → Quality-of-life (R^2^ = 0.38; *p <* 0.001)					
Constant	4.66***	0.06	63.33***	< 0.001	[4.53; 4.80]
Transactional Leadership	0.75***	0.07	10.03***	< 0.001	[0.60; 0.90]
Work Regime	0.31	0.10	3.21	0.002	[0.12; 0.51]
TransacL*WR	−0.19	0.11	−1.76	0.080	[−0.39; 0.02]

*p ≤ 0.05; ***p < 0.001. Own Source.

**Figure 4:**
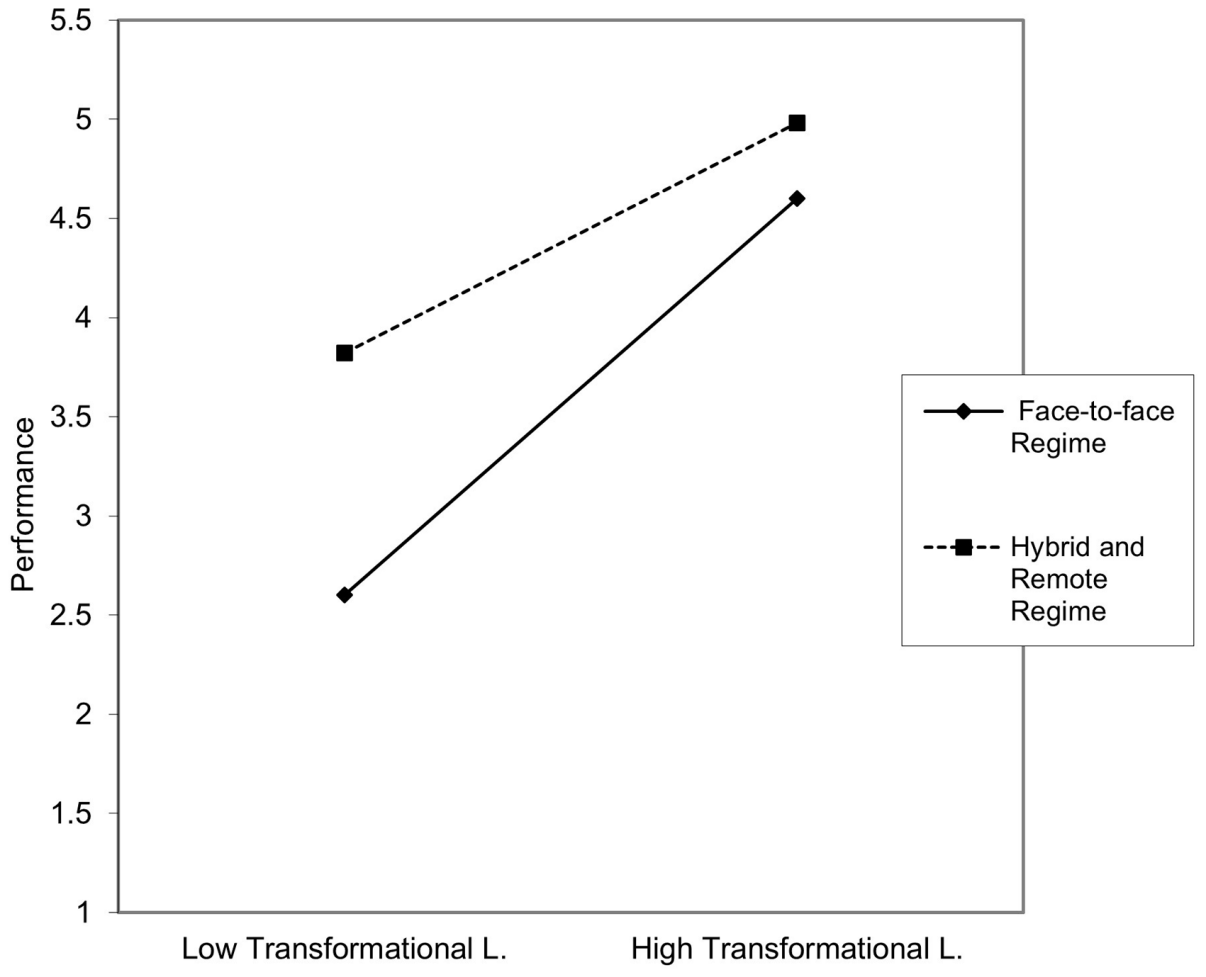
Graph showing the interaction effect of transformational leadership x work regime.

For participants in a face-to-face regime, compared to those in a hybrid or remote regime, transformational leadership becomes relevant to enhancing their perception of quality of life (Figure 4).

Hypothesis 4 was tested using a one-way ANOVA parametric test, after verifying the respective assumptions. This test was chosen because the independent variable is nominal and consists of three groups.

The work regime has a statistically significant effect on both transformational leadership [*F*(2, 228) = 4.28, *p* = 0.015] and transactional leadership [*F*(2, 228) = 7.02, *p* = 0.001] (Table 8). Participants in remote working arrangements demonstrate a perception of higher levels of transformational leadership and, above all, transactional leadership compared to participants working in face-to-face arrangements. Regarding transactional leadership, a clear pattern emerges across the three work regimes analysed: higher levels of transactional leadership in remote work, followed by hybrid work, and finally in-person work (Table 8).

**Table 8:** Effect of work regime on leadership.

Variable	One-Way ANOVA		Work Regime. A	Work Regime. B	TuKey HSD	
	F	*p*			Mean Dif. (A-B)	*p*
Transformational Leadership	4.28*	0.015	Remote	Face-to-face	0.47*	0.019
				Hybrid	0.49*	0.017
Transactional Leader	7.02**	0.001	Remote	Face-to-face	0.67***	< 0.001
				Hybrid	0.52*	0.018

*p < 0.05; **p < 0.01; ***p < 0.001. Own Source.

Next, we sought to understand whether there were statistically significant differences in leadership perception among participants in hybrid work depending on the number of days per week they work remotely. The ANOVA One-Way test indicated that there are no statistically significant differences in the perception of transformational leadership [*F*(4.75) = 1.34; *p* = 0.265] or transactional leadership [*F*(4.75) = 1.26; *p* = 0.293].

As for hypothesis 5, since it involved a mediating effect, the procedures outlined by Baron and Kenny (1986) were followed. Two multiple linear regressions were performed in two steps. In the first step, the predictor variable was introduced as the independent variable, and in the second step, the mediating variable was introduced. Considering the sample size, this was deemed the most appropriate test.

The results indicate that transformational leadership has a partial mediating effect on the relationship between work regime and quality of life. When the mediating variable was introduced into the regression equation, the work regime continued to have a significant effect on quality of life, but its intensity decreased (Table 9). The model explains 38% of the variability in quality of life (Table 9). The increase in variability proved to be significant (ΔR^2^ = 0.30; *p <* 0.001). Both models are statistically significant (Table 9).

**Table 9:** Mediating effect of transformational leadership.

Variables	Quality-of-life	
	*β* Step1	*β* Step2
Work Regime	0.28***	0.20***
Transformational leadership		0.56***
*F*	19.14***	71.59***
*R^2^*	0.08	0.38
Δ		0.30***

***p < 0.001. Own Source.

The results indicate that transactional leadership has a partial mediating effect on the relationship between work regime and quality of life. When the mediating variable was introduced into the regression equation, the work regime continued to have a significant effect on quality of life, but its intensity decreased (Table 10). The model explains 36% of the variability in quality of life (Table 10). The increase in variability proved to be significant (ΔR2 = 0.28; *p <* 0.001). Both models are statistically significant (Table 10). Table 11 summarizes the results obtained for the five hypotheses formulated in this study. As can be seen in this table, all the hypotheses formulated in this study were confirmed.

**Table 10:** Mediating effect of transactional leadership.

Variables	Quality-of-life	
	*β* Step1	*β* Step2
Work Regime	0.28***	0.15**
Transactional Leadership		0.56***
*F*	19.14***	66.75***
*R^2^*	0.08	0.36
*Δ*		0.28***

**p < 0.01; ***p < 0.001. Own Source.

**Table 11:** Summary of the results of the hypotheses formulated in this study.

Hypotheses	Results
Hypothesis 1: Leadership has a positive and significant effect on quality of life.	Supported
Hypothesis 2: The work regime has a significant effect on quality of life.	Supported
Hypothesis 3: The work regime moderates the relationship between leadership and quality of life.	Supported
Hypothesis 4: The work regime has a significant effect on leadership.	Supported
Hypothesis 5: Leadership mediates the relationship between the work regime and quality of life.	Supported

## 5 Discussion

The primary objective of this research was to investigate the relationship between leadership and quality of life, and whether this association is moderated by the work regime (remote, hybrid, or in-person). Additionally, we sought to study the mediating effect of leadership on the relationship between the work regime and quality of life.

As expected, hypothesis 1 was confirmed. Both transformational leadership and transactional leadership have been found to have a positive and significant association with the perception of quality of life, as indicated by Avolio et al. (2009), Kairupan (2023), and Hermanto et al. (2024). The higher the perception of transformational leadership and transactional leadership, the higher the perception of quality of life. These results align with the literature, not only from the perspective of Pasaribu et al. (2022) but also that of Katili et al. (2021), which suggests that leadership has a significant impact on employee commitment to a company, influencing their productivity and, consequently, their perception of quality of life.

Hypothesis 2 was also confirmed, indicating that the work regime has a significant impact on employees’ perception of quality of life, with a particular emphasis on the remote work regime, which presented the highest levels of perceived quality of life among the three regimes studied (in-person, hybrid, and remote). The results for this hypothesis are also in line with the current literature, as noted by Smite et al. (2022a), who suggest that work flexibility has become increasingly desired and even necessary for workers, leading to personal satisfaction. The remote regime, as verified in this study, is associated with a higher perception of quality of life. In line with Kausar et al. (2023), it tends to reduce worker stress while fostering higher levels of organizational commitment. Workers with greater work flexibility, especially in remote and hybrid arrangements, are likely to experience higher levels of perceived quality of life and well-being, as argued by Lundqvist et al. (2022). However, these perceptions are highly subjective to everyone, as noted by Lehto (2023). It is worth mentioning that the research by Bloom et al. (2024) is also in line with the results obtained, arguing that work flexibility is associated with a higher perception of quality of life, provided that the work regime in question is perceived as voluntary and desirable. Smite et al. (2022a) also note that the lack of flexibility has led to resistance to the face-to-face regime.

Next, hypothesis 3 was confirmed, indicating that the work regime has a moderating effect on the relationship between leadership and the perception of quality of life. Specifically, the work regime can determine whether leadership influences the perception of quality of life by subordinates to a greater or lesser extent. Here, it was revealed that there is a significant difference between the face-to-face work regime and the remote work regime. As such, leadership has a greater influence on the perception of quality of life among workers in the face-to-face work regime than in the remote work regime, demonstrating greater sensitivity, in line with the study by Lundqvist et al. (2022). Additionally, leadership effectiveness tends to depend on adaptation to the remote environment according to Coser et al. (2024). It will depend on the regime practiced (in-person, hybrid, or remote), as Kairupan (2023) argues, thus reinforcing this hypothesis of moderation by the work regime between leadership and the perception of quality of life of those being led. As a complement to this hypothesis, Wells et al. (2023) argue that leadership needs to be adjusted and adapted in remote contexts to protect the psychological health and quality of life of employees.

Hypothesis 4 was confirmed: the work regime does have a significant effect on leadership. Remote workers have a higher perception of transactional and transformational leadership, respectively, compared to those who are present at the workplace. Once again, this finding aligns with the existing literature, as noted by Kairupan (2023). Leadership should encourage and promote employee autonomy, with transformational and transactional leadership types being highlighted in the literature. It is also worth mentioning the study by Coser et al. (2024), which highlights the importance of autonomy and adaptation in remote leadership, placing greater emphasis on transformational and transactional leadership types. Dong (2023) also refers to the high perception of transactional leadership in remote working arrangements when well-adjusted to the needs of employees, with the results for this hypothesis well aligned with the current literature, given that the perception of transactional leadership was the highest. Additionally, the study developed by Tahlyan et al. (2024) recognizes that leadership requires specific competencies for remote and hybrid contexts, which in turn influences how workers perceive their leaders. Finally, the study by Smite et al. (2023) suggests that employees’ preferences for different work arrangements are linked to their perception and experience of leadership, thereby reinforcing the notion that work arrangements indeed have a significant impact on leadership.

Finally, hypothesis 5 was also confirmed and is entirely in line with the current literature, explaining how leadership acts as a mediating variable between work arrangements and quality of life. Specifically, this hypothesis suggests that workers’ work arrangements influence their perception of quality of life, taking leadership into account. In this case, both transformational and transactional leadership were shown to play a mediating role, although with variations depending on the work context. According to Clarey (2022), leading teams remotely require specific competencies, and not all leaders are prepared for this, which will ultimately impact employee satisfaction. Lundqvist et al. (2022) demonstrate that leadership performance concerning employee well-being manifests differently depending on whether the work regime is face-to-face, hybrid, or remote, thereby reinforcing the mediating role in this context. In addition, the study by Hermanto et al. (2024) demonstrates that transformational leadership has a positive impact on the quality of life at work, ultimately affecting other organizational behaviors, which supports the idea that leadership serves as a link between the work regime and perceived quality of life. The lack of competence in remote work can significantly weaken and compromise the quality of life of employees, as highlighted by Wells et al. (2023). Finally, both Siegl (2021) and Kausar et al. (2023) emphasize that leaders must adapt their practices to the reality of remote work, noting that leadership is conditioned by the work regime and how it is practiced with subordinates, considering the environment and context in question.

### 5.1 Limitations and future research

As this study’s data collection instruments were used entirely online (a questionnaire), at a distance, it may not have the same credibility as, for example, personal interviews. Although the questionnaire implemented a defense against duplicate responses, there is no guarantee that such a defense cannot be circumvented. Another possible limitation to consider is that the questionnaire was distributed through social media, which may not have reached a larger and more diverse number of participants, as not everyone has access to social media.

This research aims to contribute to future studies, deepening and expanding the topics covered here, and placing even more emphasis on the personal reasons why each worker prefers face-to-face work, as this study focused more on the reasons for working remotely.

More specifically, it is suggested that the impact of the work regime may differ between different sectors of activity, such as technology, health, public administration, etc.; investigate whether the leadership style perceived by workers is the same as that declared by the leaders themselves; understanding whether specific leadership training for hybrid/remote contexts is efficacious in improving the perception of leadership by those being led and their quality of life; studying the perception of more flexible or less flexible work regimes depending on geographical and cultural differences; and, finally, an in-depth study of work regimes is suggested, taking into account the influence of the age and generation of workers. In addition to these suggestions, a mixed-methods approach, combining quantitative and qualitative methods, is recommended to provide the study with greater depth and detail.

### 5.2 Practical implications

One of the main objectives of this study is to influence and contribute to organizations choosing better time, talent, and people management, considering the complementary topics addressed here. To provide relevant and contributory information so that various leaders can learn and become capable of working and delegating remotely whenever applicable, as well as understanding who they are delegating to, since workers do not all perform their duties in the same way, appealing to the empathy and communication skills of management towards their subordinates, as suggested by Siegl (2021).

Promote social awareness and stay up to date with technological developments in organizations, taking advantage of what they have to offer while also acknowledging their limitations and respective drawbacks. This approach enables organizations to provide a better quality of life for all employees who generate value, ultimately enhancing the overall performance of the organization. In fact, not only for the organizations themselves, but also so that managers and top leaders are aware of the vast range of options that promote people’s quality of life and results for companies by adopting sustainable practices in the long term. Another relevant point to mention is the ability that remote working provides for companies to produce value and grow, attracting talent (workers with exceptional competences and highly qualified for the tasks to be performed) that might not be possible in person and locally, as indicated by Braesemann et al. (2022), thus removing geographical barriers.

Keeping up with the evolution of standards, technology, worker needs, and current markets is essential for organizations to survive and thrive in this increasingly competitive and volatile market. If remote access to information is an asset for companies to grow, so should the discovery and retention of talent.

### 5.3 Theoretical implications

The results of this investigation reinforce the decisive role of transformational and transactional leadership styles in the perception of quality of life among an organization’s employees, as suggested by Avolio et al. (2009), who note that leadership has taken on a catalytic role in employee well-being and performance. This is further reinforced by Hermanto et al. (2024), who demonstrate the direct influence of transformational leadership on quality of life at work. The present study, therefore, contributes to existing literature by demonstrating that both transformational and transactional leadership are associated with a higher perception of quality of life among employees. This effect has become increasingly relevant in the current context of digital transition and the resulting reconfiguration of the way we work, with leaders required to possess competencies adapted to hybrid and remote environments (Clarey, 2022; Dong, 2023). Recent literature emphasizes that leading from a distance necessitates a distinct, contextual, and tailored approach to ensure the effectiveness of work performed by all employees (Siegl, 2021; Wells et al., 2023). Thus, this research contributes to the empirical validation that leadership styles are mediated and/or moderated by contextual variables, one of which is the work regime.

This study also confirms that the work regime not only directly affects the perception of quality of life, as reported by Lundqvist et al. (2022) and Bloom et al. (2024), but also plays a moderating role in the relationship between leadership and well-being, as indicated by Coser et al. (2024). This finding suggests that the influence of leadership depends mainly on the conditions under which it is exercised. The fact that the face-to-face regime reveals greater sensitivity to the influence of leadership compared to the remote regime opens up space for reformulating more traditional leadership practices. In addition, the results show that leadership acts as a kind of bridge between the work regime and employees’ perception of quality of life—which suggests that the work regime does not act in isolation in this perception, but instead in conjunction with the type of leadership practiced and the adaptation made to the remote context, as indicated by Wells et al. (2023), Kausar et al. (2023) and Lundqvist et al. (2022), which can facilitate or hinder the experience of workers, affecting their well-being and perception of quality of life. Thus, this research updates the literature in this increasingly relevant and debated context.

## 6 Conclusion

The primary objective of this study was to investigate the relationship between work arrangements, leadership, and employees’ perceptions of quality of life. The results showed that both leadership and work arrangements have significant effects on quality of life, which is a perception (and therefore subjective). Transformational and transactional leadership styles were found to have a positive association with the perception of quality of life. However, remote leadership depends on specific competencies, which will have an impact on the performance and well-being of employees.

The remote work regime translates not only into higher perceptions of quality of life but also into perceptions of higher levels of both transactional and transformational leadership. On the other hand, the face-to-face work regime revealed lower perceptions of quality of life among employees, as well as greater sensitivity to the type of leadership practiced in this context, which is reflected in a greater impact on the well-being of those being led. Work regimes (face-to-face, hybrid, and remote) have the power to influence leadership effectiveness, since this ultimately depends on the conditions in which it is exercised.

The perception of quality of life is therefore highly conditioned not only by the work regime, but also by the leadership practiced, both of which contribute to the employee experience. In contrast, the work regime will ultimately influence leadership.

The hypotheses formulated regarding leadership, quality of life, and work regime were confirmed. The results of the hypotheses align with the current literature.

This study contributes to a deeper understanding of the role of leadership in remote working contexts, highlighting the importance of adapting it to the demands of today’s increasingly digital world. By considering the needs of employees, it demonstrates that technology can be an ally in increasing individual well-being, if it is accompanied by effective leadership practices tailored to the adopted regime. Thus, workers’ perception of quality of life emerges not only because of the conditions adopted, but also from a synergy involving the context (work regime) and management capable of leading in a manner appropriate to the remote or hybrid context.

## Data Availability

The raw data supporting the conclusions of this article will be made available by the authors, without undue reservation.
